# Regulating, Measuring, and Modeling the Viscoelasticity of Bacterial Biofilms

**DOI:** 10.1128/JB.00101-19

**Published:** 2019-08-22

**Authors:** Samuel G. V. Charlton, Michael A. White, Saikat Jana, Lucy E. Eland, Pahala Gedara Jayathilake, J. Grant Burgess, Jinju Chen, Anil Wipat, Thomas P. Curtis

**Affiliations:** a School of Engineering, Newcastle University, Newcastle upon Tyne, United Kingdom; b Interdisciplinary Computing & Complex BioSystems Research Group, School of Computing, Newcastle University, Newcastle upon Tyne, United Kingdom; c School of Natural & Environmental Sciences, Newcastle University, Newcastle upon Tyne, United Kingdom; Geisel School of Medicine at Dartmouth

**Keywords:** biofilms, rheology, soft matter physics, synthetic biology, viscoelasticity

## Abstract

Biofilms occur in a broad range of environments under heterogeneous physicochemical conditions, such as in bioremediation plants, on surfaces of biomedical implants, and in the lungs of cystic fibrosis patients. In these scenarios, biofilms are subjected to shear forces, but the mechanical integrity of these aggregates often prevents their disruption or dispersal. Biofilms' physical robustness is the result of the multiple biopolymers secreted by constituent microbial cells which are also responsible for numerous biological functions. A better understanding of the role of these biopolymers and their response to dynamic forces is therefore crucial for understanding the interplay between biofilm structure and function. In this paper, we review experimental techniques in rheology, which help quantify the viscoelasticity of biofilms, and modeling approaches from soft matter physics that can assist our understanding of the rheological properties. We describe how these methods could be combined with synthetic biology approaches to control and investigate the effects of secreted polymers on the physical properties of biofilms. We argue that without an integrated approach of the three disciplines, the links between genetics, composition, and interaction of matrix biopolymers and the viscoelastic properties of biofilms will be much harder to uncover.

## Abstract (precis)

Biofilms occur in a broad range of environments under heterogeneous physicochemical conditions, such as in bioremediation plants, on surfaces of biomedical implants, and in the lungs of cystic fibrosis patients. In these scenarios, biofilms are subjected to shear forces, but the mechanical integrity of these aggregates often prevents their disruption or dispersal.

## INTRODUCTION

Polymers are ubiquitous in that they constitute the machinery of life and are found in consumer and industrial products (1, 2). Bacteria are known to secrete a variety of biopolymers that include exopolysaccharides, proteins, and extracellular DNA (eDNA) that encase the cells, resulting in the formation of “slimy” aggregates called biofilms (3, 4). The arrangements and interactions of macromolecules and cells composing the polymeric network confer upon the biofilm a dynamic architecture (5), allow it to resist invasion from external threats (invaders [6], chemicals [7], and antibiotics [8, 9]), and perform various other synergistic (10, 11) and/or antagonistic (8, 12) functions. To date, our knowledge of the genetic origins, regulation of gene expression, secretion mechanisms, and organization of various polymers within the biofilm matrix is limited (13–16), and discoveries of new biomolecules, along with their structure and biochemical implications, continually reshape our knowledge (15, 17). Recent technological advances are providing researchers with increasingly precise genetic tools in whole-genome sequencing, gene synthesis, and high-throughput screening (18–20). This opens the possibility of probing the role of single and/or multiple polymeric components and their interactions within the extracellular matrix (ECM), thereby allowing systematic investigation into the factors affecting the mechanical robustness of biofilms in new and unprecedented ways.

**FIG 1:**
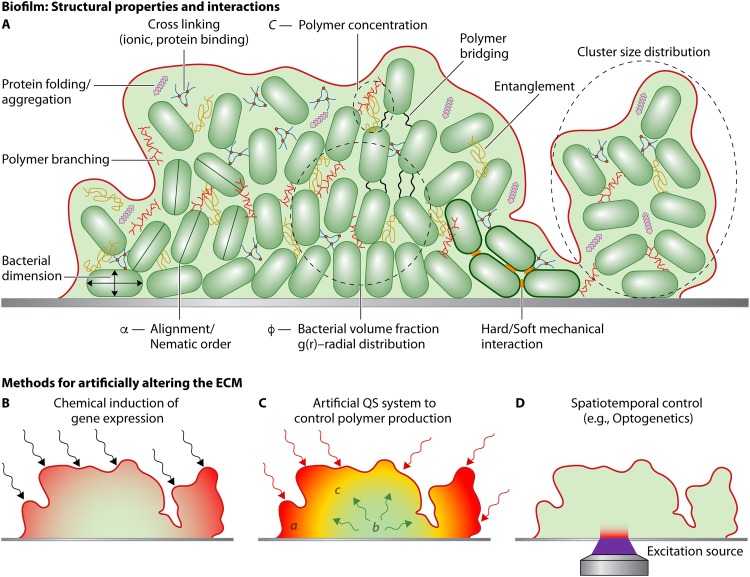
General structural components and methods for control for bacterial biofilms. (A) Overview of some of the components within a bacterial biofilm which can affect the architecture and viscoelasticity. (B) Direct induction of ECM components; chemical induction methods can be used to activate or deactivate the expression of one or more of the ECM components. (C) Synthetic QS-based control potentially allows different ECM components to be expressed based on the population densities of different strains. QS also allows for signal amplification through the biofilm structure, thereby complementing direct induction (as seen in panel B). (D) Optogenetic control mechanisms can be used to direct the expression of certain structural components within a growing biofilm at precisely controlled locations.

Upon application of stress, a biofilm exhibits both elastic and fluid-like behavior, a time-dependent response known as viscoelasticity. Rheology is the study of such viscous and elastic responses in materials and seeks to decipher the changes in the underlying structure due to the application of forces. Biofilms are unique rheological systems because they comprise living cells and a dynamic extracellular matrix. ECM secretion is driven by the interplay between gene expression and environmental conditions (21) resulting in compositional and spatial heterogeneity. The constituent macromolecules self-assemble (22) via polymer interactions such as entanglement, protein binding, and cross-linking to form a transient stress-bearing structure (Fig. 1A) (16, 23, 24). Recent studies have shown that ECM constituents, such as proteins, eDNA, and polysaccharides, dictate biofilm architecture as well as matrix viscoelasticity. However, there is a lack of understanding of the structural rearrangements, cross-linking, and behavior of matrix biopolymers under large shear forces. Modern rheological techniques like large-amplitude oscillatory shear (LAOS) and optical tweezing (OT) allow us to record rheological signatures at a variety of strain amplitudes with high temporal fidelity, thereby allowing us to advance our understanding of interlinking or entanglement of cells and extracellular polymers from a mechanics viewpoint.

**FIG 2:**
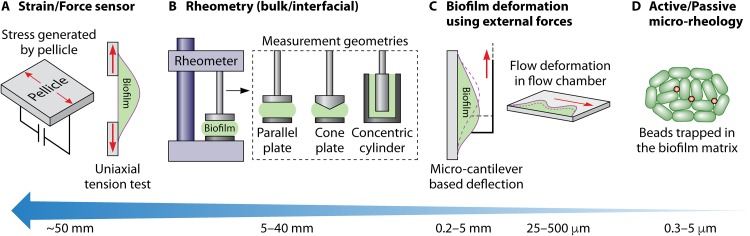
Techniques for measuring rheology of biofilms arranged in decreasing order of the length scale. (A) Extension/compression tests of biofilms/pellicles using force sensors. (B) Bulk/interfacial rheometry performed using a rheometer and the different kinds of measurement geometries that can be used in a rheometer. (C) Deformation of biofilms within fluidic chambers using flow forces or by using a microcantilever. (D) Microrheology technique in which beads are trapped within biofilm and the motion of the beads is driven either by thermal fluctuations or through an external force.

A variety of mechanical (25) and spectroscopic (26) techniques exist for characterizing the viscoelasticity of biofilms at multiple length scales (27) (Fig. 2). Biofilms growing under different environmental conditions are known to exhibit large variations in viscoelasticity (28). Coupled with the complexity arising from a multiplicity of measurement tools at different length scales, the need for standardized mechanical measures has been highlighted (29). Matrix viscoelasticity is known to confer protection against physical and chemical perturbations (30) and has also been attributed a role in the virulence of Pseudomonas aeruginosa (31). While the literature alludes to the structural role of biopolymers (28, 30), a systematic discussion on deciphering their roles from a molecular biology and physical viewpoint is still lacking. Boudarel and coworkers (29) called for a standardization of methods for characterizing and measuring biofilm structure; however, we would go further than this. We argue that if modeling approaches from soft matter physics are employed alongside data from experimental rheology techniques, this would improve our ability to quantify and characterize biofilms and their structures. Modeling approaches from soft matter physics, in essence, would simplify the complexity of biofilms, treating them as materials that can be described by a set of physical parameters. Here, we review approaches from synthetic biology (SynBio), experimental rheology, and soft matter physics. We focus on where these methods have revealed new insights into biofilm structural properties and where the techniques have begun to be used together to form new multidisciplinary approaches to address questions in biofilm research.

## GENETIC TOOLS FOR MANIPULATING THE VISCOELASTICITY OF BIOFILMS

Early research into the genetics of biofilms was predominantly based on screening mutant libraries for biofilm deficiency (32–34). Molecular approaches have enabled the creation of strains, where overexpression or deletion of particular matrix component affects the biofilm structure and viscoelasticity. Experimentally controlling the spatiotemporal dynamics of polymer secretion remains challenging because traditional overexpression and deletion strains cannot be modulated *in situ*. SynBio has been widely used in microbiology to produce novel metabolites, nanomaterials, and biosensors. However, the use of SynBio tools in creating engineered biofilm-like materials and in understanding rheology of biofilms is limited (35–37). The following section summaries the various SynBio techniques that could be employed to manipulate the secretion of ECM components and the type of control each method offers (Fig. 1B to D).

### Chemical induction.

Owing to the multiple regulatory, synthesis, and posttranslational steps involved in the ECM assembly processes, engineering a phenotype beyond on/off remains challenging. Several groups have demonstrated the advantages of modulating the levels of expression of individual polymeric components using standard molecular biology approaches (38). For example, chemically induced gene expression (Fig. 1B) has been used in studies concerning the spatial structuring of both Vibrio cholerae and P. aeruginosa biofilms. These techniques have assisted in revealing the role of protein CdrA, which mediates cellular packing and cell aggregation in P. aeruginosa biofilms in the absence of polysaccharides (17). A CdrA-rich biofilm matrix has been found to have a compact architecture, and cross-linking of CdrA with Psl (one of the polysaccharides produced by P. aeruginosa) has been found to confer protection against proteolysis. Hartmann et al. (39) used single-cell microscopy in conjunction with the control of RbmA (a mediator of cell-cell interaction) to understand how RbmA expression influenced cellular positioning in the extracellular matrix of V. cholerae. By measuring structural parameters such as intercellular distances and local density of cells, they were able to derive a theoretical model (that considers interaction potential between cells) to describe the microstructural architecture of the biofilm. Artificially controlling the levels of cyclic di-GMP (c-di-GMP), a master regulator of biofilm formation in a number of bacterial species (40) using light-responsive promoters has been used to assert temporal control over P. aeruginosa biofilm formation (41). Advancements in understanding the organization of c-di-GMP networks open the door for producing engineered strains with increasingly precise regulatory control (42). For instance, the ability to construct strains where the retention and release of surface-bound proteins could be controlled by c-di-GMP was recently demonstrated in the Lap system of Pseudomonas fluorescens (43). These approaches could be used to study the roles of individual ECM components on cell-cell interactions and the rheological fingerprint of growing biofilm clusters.

### Quorum sensing-based control.

An alternative approach to exert control over ECM components would be engineered quorum sensing (QS) systems (44, 45). QS is used to coordinate inter- and intraspecies phenotype changes based on population density. QS plays a role in regulating biofilm formation, surface and secreted virulence factors, community interactions, and dispersion across many bacterial species (46, 47). Rational bottom-up design using laboratory and modeling approaches has also resulted in the design of ultrasensitive QS switches that can tightly regulate gene expression (48) and force coordinated behavior between strains. These systems can mimic simple transistor switches (Boolean logic) which have allowed investigators to exert sophisticated control over polymer secretion and competition dynamics (49, 50). Such systems have been used in Komagataeibacter rhaeticus, where cellulose expression was repressed over a 10-fold range by the QS molecule acyl homoserine lactone (AHL) via a small RNA (sRNA) repression mechanism (51). However, these approaches are dependent upon the diffusive transport of QS autoinducers and therefore lack spatial control. By temporally regulating polymer expression at different times, heterogeneous environments can be created, resulting in a composite-like material (Fig. 1C). The structure and rheological heterogeneity of such materials can then be studied using microrheological techniques, such as optical tweezing.

### Spatiotemporal control.

Depending on the species, stage of growth, and environmental stresses, biofilms can develop into heterogeneous structures. A biofilm’s local rheology varies with spatial location and temporal dependence of the polymeric secretions. Therefore, controlling the initial spatial distribution and spatiotemporal secretion of polymers (Fig. 1D) in developing biofilms would be advantageous. The ability to synthetically differentiate cells within a population based on location has recently gathered attention, as it can help in the production of biological materials with microscale patterns (52). A number of methods have been used to bind microbes to specific locations on a two-dimensional (2D) surface (53). These include using surface-bound antibodies and binding proteins specific to individual strains, as well as chemically binding DNA to sugars on the microbial surface (54). The microbially bound sequences then hybridize to a corresponding sequence which can be arrayed in a predetermined pattern on a 2D surface. A toolbox for preprogramming cell-cell adhesion and manipulating microbes into predetermined structures without the need for surface binding has also recently been developed (55). Methods of *in situ* precise spatiotemporal control over gene expression have been achieved using optogenetics to induce formation and control the shape of P. aeruginosa biofilms (41).

Theoretically, the use of SynBio tools could allow the programming of a microbial population where strains are organized into precise locations on a surface before being allowed to generate biofilms of different compositions. Polymers could then be induced at different times or locations across a homogenous or heterogenous population to form precisely controlled microscale structures. Such fine-tuned spatial and compositional control would enable experimenters to use rheological methods, such as optical tweezing and LAOS, to perform experiments characterizing both structural and micro- or macrorheological changes. In a very recent example, Bacillus subtilis (56) was engineered as a living biomaterial by linking secreted TasA amyloid monomers to functional proteins, including pollutant degradation enzymes. The modification of TasA resulted in biofilms with lower viscoelasticity; as a result, the engineered biofilms could be three-dimensionally (3D) printed into predetermined shapes. This idea demonstrates the unique ability of SynBio tools for designing artificial living materials where the rheological fingerprint could be fine-tuned artificially, thereby allowing researchers to study the roles of individual polymers more precisely than before. For a recent perspective on engineered living biomaterials (ELMs), we recommend a paper by Gilbert and Ellis (52).

## EXPERIMENTAL TECHNIQUES TO QUANTIFY THE RHEOLOGY OF BIOFILMS

Owing to the variability in composition, cultivability, and stiffness, a variety of multiscale techniques have been used to measure biofilm rheology (Fig. 2). At the scale of few centimeters, bulk elastic moduli have been determined by performing uniaxial compression (57) or tension (58) tests on biofilms. Internal compressive stresses generated by a growing pellicle have also been measured using a customized apparatus (59, 60) (Fig. 2A). Dynamic oscillatory (61) or interfacial rheology (62) tests use a rheometer fitted with different measurement geometries (Fig. 2B) and have been used to probe the elastic and viscous moduli at the centimeter scale. The technique has revealed that the variation of the moduli span orders of magnitude among different species of microbes (63–66). The effects of genetic modification and chemicals, such as divalent or trivalent cations and surfactants (31, 67–72), in altering biofilm rheology have also been quantified using a rheometer. Imaging techniques that rely on measuring the deformation of biofilm through application of fluid shear (73, 74) have shown the transition in behavior from viscoelastic solid to liquid-like beyond a threshold stress (75) and have also demonstrated stiffening of biofilms due to large forces (76). Deflection of biofilms using a microcantilever (77) has revealed an increase in strength of biofilms when the force is applied at a high strain rate (78) (Fig. 2C). At microscale, a variety of active and passive microrheology techniques use micrometer-sized beads trapped within the biofilm network to probe the rheological characteristics. Passive microrheology uses the ambient energy in the surrounding environment, which results in Brownian motion of the beads, while active microrheology uses an external driving force (light beam or magnetic field) to manipulate the motion of the micrometer-sized beads within the medium. Various microrheology techniques (Fig. 2D), like particle tracking rheology (79–82), diffusing wave spectroscopy (22, 83), optical tweezing (84), and magnetic tweezing (85, 86), have been used to investigate how architecture, environmental fluctuations, and genetically mediated changes in ECM composition result in rheological heterogeneity in different species of biofilms.

**FIG 3:**
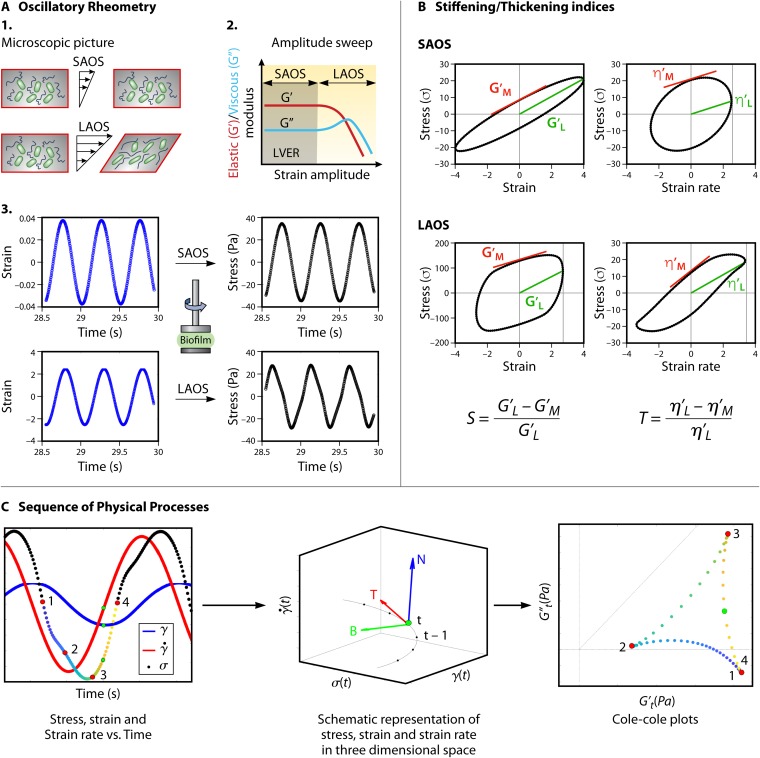
(A1) Microscopic picture of biofilms. In small-amplitude oscillatory shear (SAOS), the material structure remains intact, whereas the application of large-amplitude oscillatory shear (LAOS) causes the material to irreversibly deform. (A2) Amplitude sweep showing the variation of elastic *G*′ and viscous moduli *G*″ as a function of strain amplitude. (A3) Application of SAOS results in a sinusoidal stress output indicating linearity of the material, while LAOS results in stress output that is nonsinusoidal, indicating a nonlinear response. (B) Representative Lissajous-Bowditch plots in the SAOS and LAOS regime, the small/large strain moduli for those plots and the formulae to calculate stiffening (*S*) and thickening (*T*) indices. (C) In SPP, stress is plotted as a function of strain and strain rate in 3D space. At each of the successive points, the transient moduli [*G*′*_t_*(*t*) and *G*″*_t_*(*t*)] are used to generate Cole-Cole plots, which can be used to study stiffening/thickening of biofilms.

Most techniques that are applied to measure biofilm viscoelasticity use small strains in the linear viscoelastic region, which means that the initial biofilm structure remains preserved (Fig. 3A1). However, in both natural and artificial environments, biofilms can experience large forces or rapidly applied loads, causing structural rearrangement that results in a nonlinear material response (76, 78). The nonlinear responses manifest as stiffening or softening and thickening or thinning. The emergence and magnitude of each characteristic behavior are dependent on the breakage of bonds, cross-links, and entanglements between a variety of polymeric components (Table 1) and the spatial organization of biofilm architecture (Fig. 1A). Rheology measurement techniques, like LAOS and optical tweezing microrheology (OT-μR), described in the following sections, allow us to probe both the linear and nonlinear and steady-state and time-dependent response of biofilms, with a focus toward understanding the interactions between the components of a biofilm’s matrix.

**TABLE 1:** Proteins and polysaccharides present in the ECM of different species of biofilms and their structural role

Species	Component	Polymer type	Function (reference)
E. coli	Cellulose	Polysaccharide	Architectural element in biofilms, together with CsgA, contributes to elasticity (139)
	Curli/CsgA	Protein	Constituent of curli fibers, forms composite with cellulose (139)
	Curli/CsgB	Protein	Nucleates polymerization of curli fibers (14)
	Antigen43	Protein	Promotes cell-cell adhesion (14)
	FliC/MotA	Protein	Controls wrinkle formation (139)
P. aeruginosa	Pel	Polysaccharide	Scaffold for the biofilm, maintains intercellular interactions (16)
	Psl	Polysaccharide	Initiates biofilm by modulating cell-surface and cell-cell attachment (140, 141)
	Alginate	Polysaccharide	Overproduction results in mucoid phenotype and alters the viscosity of biofilm (72)
	CdrA	Protein	Controls cellular packing and protects matrix components from proteases by linking with Psl (17)
B. subtilis	Unnamed	Polysaccharide	Part of matrix, exact composition unknown (14)
	BslA	Protein	Forms hydrophobic coating at the periphery of the biofilm and contributes to the rugosity (14)
	TasA	Protein	Helps in formation of amyloid-like fibers and is responsible for rugosity (14).
	TapA	Protein	Facilitates TasA fiber assembly and attachment (14)
V. cholerae	*Vibrio* polysaccharide (VPS)	Polysaccharide	Scaffolding material of the extracellular matrix (70)
	Bap1	Protein	Helps in cell-surface adhesion and cross-links with VPS, controls elasticity of pellicles (70)
	RbmA	Protein	Connects neighboring cells by dimerizing with VPS (70)
	RbmC	Protein	Cross-links with VPS and helps in cell-surface adhesion (homologous to Bap1) (70)

### Rheometer operation.

Rheometers are versatile instruments for studying soft matter systems like colloids, suspensions, and gels. In the past few decades, they have also become invaluable for investigating the viscoelasticity of biofilms. The notable components in a rheometer consist of a fixed flat-bottom plate on which the sample is usually placed and a top geometry that can be bought in contact with the sample to apply a controlled amount of deformation/force (Fig. 2B). Rotational rheometers rely on the application of controlled oscillatory shear stress (σ) or strain (γ) on the biofilm sample and recording the material response. By knowing the amplitude (γ*_o_*) and frequency (ω) of the input strain waveform, as well as the amplitude (σ*_o_*) of the output stress signal with respect to time (*t*) and the phase lag (δ), one can use equations described by Ferry (87) to calculate the elastic and viscous responses of the material. The measures commonly known as the elastic modulus (*G*′) and loss modulus (*G*″) describe the rigidity and fluidity of the material. The calculation of elastic and loss moduli assumes that infinitesimal strain is applied on the material so that both input and output waveforms are sinusoidal (Fig. 3A3). The most common test performed in rheology is known as amplitude sweep, and it involves subjecting the material to sinusoidal strain waveforms of increasing amplitude (keeping the oscillation frequency constant). Figure 3A2 shows a typical result of amplitude sweep for biofilms. The elastic and viscous moduli exhibit constant values at small strain amplitudes; this regime is typically referred to as the linear viscoelastic region (LVER). In the LVER, the input (strain) and output (stress) signals remain sinusoidal, describing a linear response of the material. As seen in Fig. 3A1, the material structure remains completely intact due to the application of small strain. At larger values of strain (beyond the LVER), the stress waveform is no longer a sinusoid. In this nonlinear region, polymer entanglements break, material structure gets rearranged, and local stiffening/softening or yielding of material can occur depending on the magnitude of the input strain. Since the linear viscoelastic analysis does not take into consideration the shapes of the stress waveforms, important information describing the above-mentioned physical processes occurring in the material is lost. In the following sections, we describe the techniques of large-amplitude oscillatory shear (LAOS), which analyses the shape of waveforms to provide some measures of quantifying the nonlinear rheological behavior occurring within the materials.

### LAOS.

**(i) Lissajous-Bowditch plots and Chebyshev polynomial analysis.** An increase in the magnitude of strain amplitude beyond the LVER results in the stress waveform transitioning from a sinusoid to nonsinusoidal shape (Fig. 3A3). A geometrical way of looking at these nonsinusoidal waveforms is to eliminate the parameter time (*t*) in strain (γ) versus time (or strain rate [γ̇] versus time) and stress (σ) versus time plots, and to look at the plot of stress (σ) versus strain (γ) (or stress [σ] versus strain rate [γ̇]). These plots of (σ) versus (γ) (or [σ] versus [γ̇]) are known as elastic (or viscous) Lissajous-Bowditch (LB) plots, respectively, and provide a geometric way of describing the state of the material. The elastic LB plot takes the shape of an ellipse, and the viscous LB plot takes the shape of a circle in the linear regime, as seen in Fig. 3B. At large strain amplitudes, the LB plots can exhibit parallelogram-like or sigmoidal shapes depending on the extent of nonlinearity of the material (Fig. 3B). The material state based on the shapes of LB plots is best described through numerical values of intracycle strain stiffening (*S*) or intracycle shear thickening (*T*) indices, which are the ratios of minimum and large strain moduli (Fig. 3B); minimum strain modulus (*G*′*_M_*/η′*_M_*) is defined as the slope of the tangent to the elastic/viscous LB plots at zero strain, and large strain modulus (*G*′*_L_*/η′*_L_*) is the slope of the line joining the origin to maximum stress (Fig. 3B). Depending on the shapes of the LB plots, the values of *S* and *T* can go either positive or negative, with *S* > 0 (*T* > 0) indicating intracycle strain stiffening (shear thickening) and *S* < 0 (*T* < 0) indicating intracycle strain softening (shear thinning). These measures (*S, T*) at various points in the Pipkin diagram (strain amplitude versus frequency) allow one to generate rheological fingerprints. Rühs et al. used similar concepts for studying the pH-mediated stiffening of β-lactoglobulin fibrils, peptides, and monomers using an interfacial rheology setup (88). By generating fingerprints of stiffening index, they found that maximum stiffening occurs in β-lactoglobulin fibrils at intermediate pH and attribute this to the formation of multilayer aggregates. A similar interfacial rheology setup was also used to quantify the differences in stiffening indices (based on elastic LB plots) of Pseudomonas putida pellicles at various stages of development during a 60-h growth period (89).

Another approach to analyzing the resulting nonsinusoidal stress waveforms was developed by Ewoldt et al. (90) and is implemented in the freely available software MITlaos (91). The technique approximates the shape of nonsinusoidal waveforms using mathematical functions (subject to mathematical assumptions [92]) like Chebyshev polynomials and calculates the contributions of first-, third-, and fifth-order harmonics to determine the elastic and viscous components of stresses. The first-order harmonic describes the linear response of the material and gives the same measures as the elastic and loss moduli. A positive value of the third-order elastic or viscous coefficient (*e*_3_ or *v*_3_, respectively) indicates stiffening or thickening, while a negative value indicates softening or thinning, respectively. A detailed description on the calculation of these measures can be found in references 93 and 94. This technique was recently applied to single- and double-stranded DNA solutions, which revealed that the double-stranded DNA solution showed persistent intracycle stiffening for strain amplitudes greater than 100% and shear thinning behavior across all strain amplitudes (95). However, single-stranded DNA exhibited a complex mixture of stiffening/softening or thickening/thinning behavior at various strain amplitudes. Pronounced strain stiffening characteristics have also been observed for the mucus of gastropods that impose large oscillatory strain while moving on surfaces (96). Extracellular components, like eDNA, form an essential part of P. aeruginosa, Myxococcus xanthus, Streptococcus mutans, and various other biofilms. eDNA is known to cross-link with polysaccharides to provide structural support to the biofilms (97). It is also suspected to increase the microcolony strength (98, 99) and increase the viscoelastic relaxation times in biofilms (64). If the cross-linking between these polymers results in stiffening/thickening, the LAOS measures described above can help decipher the nature of mechanical interactions. Also, by plotting the intracycle stiffening (*S*) and intracycle thickening (*T*) indices in the Pipkin diagram, the limits of environmental, chemical, or pH-based fluctuations that these biofilms can withstand mechanically can be determined. A similar polymeric interaction-mediated change in viscoelasticity occurs in P. aeruginosa biofilm, wherein the matrix protein CdrA cross-links with Psl to confer protection against proteases (17, 27). LAOS can be a useful tool for probing such polymeric interactions that cause stiffening or thickening of the matrix or to examine changes in the nonlinear behavior of biofilms formed by deletion mutants of Psl or CdrA in P. aeruginosa.

**(ii) Sequence of physical processes.** One of the limitations of Chebyshev polynomial analysis is the requirement for steady-state full-cycle stress and strain waveforms (Fig. 3A3) which are used to calculate an average value of higher-harmonic components. In addition, the mathematical assumption behind Chebyshev polynomial analysis (92) can be violated for a variety of samples that are tested in the laboratory (100). To overcome these challenges, a new method known as sequence of physical processes (SPP) (101, 102) was proposed. SPP uses a differential geometry-based approach and represents the stress, strain, and strain rate (derivative of strain with time) as independent axes in a three-dimensional space, as seen in Fig. 3C. Using the mathematical relations described in reference 103, each point along the oscillation cycle can be used to compute the transient moduli, i.e., *G*′*_t_*(*t*) (transient elastic modulus) and *G*″*_t_*(*t*) (transient loss modulus) as a function of time. A parametric plot of *G*′*_t_*(*t*) and *G*″*_t_*(*t*) allows material response to be represented using Cole-Cole plots (Fig. 3C), from which stiffening, softening, thinning, and thickening dynamics can be understood. Figure 3C describes the series of physical processes a material goes through in response to an applied strain waveform. The first step involves a slight thickening along with softening, followed by a large thickening and stiffening event; finally, the material exhibits thinning with little change in the transient elastic modulus. This series of processes can also be phenomenologically understood in terms of stretching, breaking, and reformation of nearest-neighbor cages or bonds, a framework commonly used to describe the microstructural response in colloidal suspensions and gels (104). Recent experiments with biofilms produced by matrix-producing and matrix-nonproducing strains of V. cholerae exhibit a 3-fold difference in viscosity. The motion of tracer beads in the nonproducing strain has been found to exhibit caging-like dynamics owing to the dynamic formation and breakage of cellular clusters arising due to cell death (105). The method has also been applied successfully in explaining the dynamics of biological fluids, such as human blood and hyaluronic acid (106, 107). SPP allows temporal representation of biofilm yielding, perhaps enabling the detection of subtle genotypic changes influencing cell-cell adhesion and ECM-cell interaction.

### Optical tweezing microrheology.

The development of optical tweezers (OT) is a Nobel Prize-winning technique (108). Within biological systems, OT have been used to measure stretching profiles of DNA, determine the binding strength of actin to cross-linking polymers, and measure the deformability of red blood cells (109), the cytoskeleton (110), and cell membranes (111). OT rely on the use of a highly focused laser beam to provide a force that is able to manipulate micrometer-sized particles, either by attracting or repelling them. For reviews on the operation, setup, and physics, see references 112 and 113, and for the application of optical tweezing microrheology, see reference 114.

**FIG 4:**
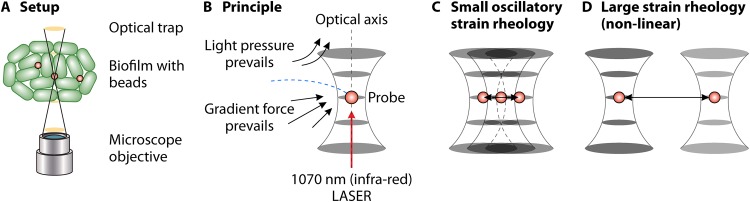
Schematic of working. (A) Schematic of an optical tweezer on a microscope. (B) Forces experienced by the particle in an optical trap. (C) Linear microrheology carried out using optical trapping to oscillate a bead. (D) Nonlinear microrheology, moving the trapped bead with a large strain out of the range of linear viscoelasticity. The traps can also be turned off, and recovery of the material can be measured by tracking the beads.

Advances in microrheology have led to the application of both optical and magnetic tweezers to measure the viscoelasticity of complex fluids using active forces in a noninvasive manner (115). Active microrheology (like OT) involves driving microspheres through a material, usually in a sinusoidal manner (by using a sensitive piezo stage or a piezo mirror) and measuring the mechanical response. Trapped beads can be controlled to nanometer and millisecond precision (114, 115), allowing the forces to be measured with subpiconewton accuracy. By controlling the strain amplitude and the frequency, both the linear and nonlinear material responses (Fig. 4) can be recorded, and the material measures can be calculated using the relationships described in reference 114. OT systems are calibrated by measuring trap stiffness, which depends upon particle size, the laser power that is reaching the sample, and the wavelength of the trapping laser. Values can range from 0.1 to 4,000 pN μm^−1^ W^−1^ for the silica and polystyrene microparticles (116) commonly used in OT-μR. OT-μR in conjunction with click chemistry (117) (using functionalized beads) can allow one to probe the rheological dynamics of individual polymers or their interactions with other molecules within the biofilm with high spatial resolution, thereby making it a useful tool in probing the heterogeneity of the biofilm matrix. Applications of OT-μR in measuring the viscoelasticity of biofilms are discussed below.

Osterman et al. (118) carried out one- and two-particle OT-μR to measure temporal changes in the viscosity of bacterial cultures and showed that polymeric constituents play a subtle role in changing the viscoelastic characteristics of media at different stages of growth. Sjojković and coworkers (119) demonstrated the suitability of OT-μR toward characterizing the interactions between DNA and levan, which phase separate when mixed together. Levan is a natural polysaccharide known to be important in stabilizing biofilm formation (120). Macroscopic rheometer measurements indicated negligible interaction between levan clusters and DNA; however, OT-μR showed otherwise. The result was confirmed by the addition of DNase, which caused levan aggregates to disperse, indicating the ability of OT-μR to probe more subtle interactions between the polymeric components of the matrix. The sensitivity of the OT was also used to understand the early mechanical coupling between bacterial cells in cultures. Sretenovic et al. (84) used optical tweezers to move bacterial cells and found that they could be tethered over distances ranging from 60 to 140 μm, indicating the formation of loosely connected aggregates. Transmission electron microscopy (TEM) and scanning electron microscopy (SEM) imaging confirmed that ECM did indeed bind the cells together, and the mechanical coupling varied between the species. The tweezers were also used to perform active microrheology measurements on the cultures, revealing that the extracellular matrix material is viscoelastic. As with all active-matter rheology experiments, one should be careful that the measurement time scale is sufficiently small so that system characteristics do not change (121) over the measurement period. The activity within biofilms can be minimized by using appropriate buffer solutions allowing the measurement time scales to be increased.

## MATHEMATICAL MODELING APPROACHES FROM SOFT MATTER PHYSICS

Mathematical models that describe biofilm rheology are important because they allow one to capture a wide spectrum of behaviors using minimal variables. Carefully constructed models can account for not only for polymeric interaction-mediated effects (like softening and thinning) but also the effect of extraneous factors like metal ion-mediated cross-linking of the matrix, etc. Until recently, biofilms have been described as continuous materials which can be considered to consist of springs and dashpots that capture the macroscopic elastic and viscous behavior. The springs or dashpots can be connected in series (Maxwell model) or parallel (Kelvin-Voigt model) or more complex arrangements (Burger/Jeffreys models) and have been extremely successful in capturing the creep and relaxation behaviors of biofilms (63, 65, 68, 122–124). In addition, the nonlinear Burger model (122), linear springs (125), and phase-field models (126, 127) have been used to describe the deformation behavior of biofilms subjected to fluid shear. However, most of these models only describe the linear response of biofilms while ignoring the details of polymeric interactions in the biofilm matrix. The following section describes two modeling approaches from soft matter physics that can be used to capture the details of the nonlinear rheological behavior in biofilms.

### Discrete model(s) with interaction potential.

**FIG 5:**
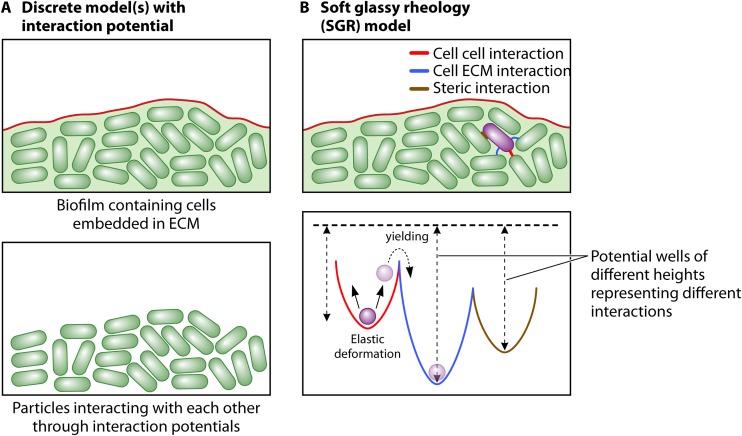
Modeling approaches that can capture microstructural and rheological details of biofilms. (A) Discrete model(s) with interaction potential. Top, structure of biofilms in which cells are embedded within the ECM. Bottom, simplified description, in which only the positions of the bacteria are taken into account and a potential function is used to describe their interactions. (B) Soft glassy rheology model. Top, the bacteria interact not just with each other but also with the ECM. Factors like steric interactions, charge effects, etc. can play a role in the biofilm rheology. Bottom, for modeling purposes, each of the different interactions can be thought of as a potential well with varied height.

Until recently, the ability to acquire precise *in situ* microscale biofilm structural parameters was limited. However, the advent of single-cell resolution microscopy platforms and sophisticated image segmentation algorithms has enabled the calculation of a plethora of structural parameters (128). By taking a minimal number of experimental parameters, like bacterial number density and pair correlation function (describes the probability of finding another cell within a specified distance), the macroscale rheology of a biofilm system can be computed. One such model is called the point process model (129), and it has been used to evaluate the effect of microscale cellular position and bacterium-bacterium interaction on the bulk rheology of biofilms (Fig. 5A). Implementation of these models has generated insights into how microstructural variability increases macroscopic strength, and rheological predictions from the model have matched closely with the results from experiments (129). Incorporating additional complexity, by accounting for the contribution of ECM components within the point process theory, can be made possible by using network models (130, 131). For example, to model the role of polymeric components on the microscale structure of V. cholerae biofilms, a pairwise potential model was used. The potential function incorporated terms which accounted for cell-cell- and cell-ECM-mediated repulsive or attractive interactions. The model was able to describe the structural rearrangement of biofilms in response to fluid shear and found good agreement with previous experiments (39). These models in conjunction with SynBio tools, which offer spatiotemporal control of polymer production, can help understand how local variances in structure alter the micro- and macrorheology and stability of biofilms.

### Soft glassy rheology model.

The soft glassy rheology (SGR) model is phenomenological in nature and has been used to describe the rheology of glasses, foams, and emulsions (132). The model has been recently adapted to include active force generation and applied to active-matter systems, such as eukaryotic cells that contract and relax via polymerization and depolymerization of actin and myosin (133). The central assumption behind the model is that the material consists of infinite mesoscopic elements, with each element being linked to others through weak interactions. The strength of the interactions can be thought of as a particle in a potential well where the depth of each well is different (Fig. 5B). Each well (having a different depth) represents different interactions within biofilms, like binding energies of polymers to each other, cross-linking strengths, steric effects, charge-mediated interactions, etc., that occur in the system under consideration (e.g., biofilms and eukaryotic cells). The mesoscopic elements cannot escape the well because of thermal fluctuations only and need significant energy to overcome the potential barrier. The motion within the wells is representative of elastic deformations in the material. And, as the element escapes the well (due to increased energy), yielding occurs and energy is dissipated as heat. This theoretical framework assists in the description of structural transition events, like elastic deformation, yielding, and reformation of bonds, akin to SPP, thereby allowing for comparisons between experiments and models. Advanced models, like glassy worm-like chain, stiff filaments with flexible linkers (133) that provide accurate description of geometric interactions between the various polymers, can also be employed to study the polymeric interactions within the ECM. Some of these models have already been used to understand stiffening, power law rheology, and changes in terminal relaxation within eukaryotic cells (133).

## DISCUSSION

In summary, we have discussed various tools from SynBio, experimental rheology, and modeling techniques that can be employed together to address multidisciplinary questions in the area of viscoelasticity of biofilms. These physical approaches allow bacterial biofilms to be considered as living colloidal gels, wherein the cell secretes a number of polymeric substances which are regulated by gene expression and the genotype of the cell. The production of multiple polymeric components might be a bet-hedging strategy employed by bacteria to ensure survivability in unpredictable environments. It is also starting to become clear that interactions between polymers are a critical determinant of the rheological behavior of biofilms and their functionalities (17, 31). SynBio tools could play a crucial role in deciphering such interactions by controlling the levels of expression of the various polymers. For example, cross-linking between anionic eDNA and cationic polysaccharide Pel is proposed to confer P. aeruginosa biofilms their structural stability, but the exact details of the interaction and rheological ramifications remain unclear. A combination of polysaccharide-protein interactions in P. aeruginosa biofilms (17) also could be investigated, where focus lies on characterizing matrix viscoelasticity, as well as functionality and understanding the trade-off between the two. The active rheological techniques of LAOS and optical tweezing have an important role to play in constructing rheological fingerprints, which could lead to a more robust understanding of the matrix polymers (or their interactions) which affect the architecture and mechanics of biofilms. A similar confluence of a few of the above-mentioned techniques was employed by Huang et al. (56) to design biofilms with tunable mechanical characteristics that could be 3D printed and possess pollutant-degrading functionalities.

In all the above-mentioned situations, rheological modeling approaches (from soft matter theory) have a major role to play in defining and testing structure-function relationships. Experimental macrorheology tools provide the ability to record signatures with high throughput and fidelity that can be indicative of stiffening, cross-linking, stress overshoot, etc. However, these tools cannot directly visualize the polymeric interactions. In these scenarios, by employing an SGR model and drawing analogies to similar colloidal systems, numerical tools can present a picture of the molecular interactions and their effects on bulk biofilm viscoelasticity. Machine learning tools applied to materials science (134–136) are set to accelerate discoveries in this field and open up the possibility of designing artificial biofilms in conjunction with environmental functionalities (56, 137). A confluence of ideas and techniques from all three different disciplines is crucial to answering fundamental questions about biofilm structure-function relationships, and for the development of biofilm-inspired synthetic biomaterials.

## APPENDIX

### GLOSSARY

amplitude sweep: A plot showing the variation of elastic and loss modulus versus strain amplitude.
Boolean logic: Simple “and” and “or” gates that can be genetically encoded using bottom-up design approaches.
Chebyshev polynomials: A class of polynomials with special properties that can be used to approximate various functions.
creep: The slow progressive deformation of material under a constant stress.
elastic modulus: The elastic-like behavior (ability to store and release energy) of a material.
harmonics: A signal whose frequency is an integer multiple of the frequency of a reference signal. Harmonic analysis refers to a mathematical technique that deals with representation of complex waveforms using a combination of some basic waves.
intracycle shear thickening: Increase in loss modulus with increase in strain rate (138).
intracycle strain stiffening: Increase in elastic modulus with increase in strain.
loss modulus: Flowability (ability to dissipate energy as heat) of the material.
linear viscoelastic region: A region in the amplitude sweep where the elastic and loss modulus remain constant.
Pipkin diagram: The material response in 2D space; one of the axes is applied frequency, while the other axis is the magnitude of strain amplitude.
transient moduli: The instantaneous elastic or viscous response of a material. Calculated using the formulae described in reference 103.
